# Relationship between long non-coding RNA PCAT-1 expression and gefitinib resistance in non-small-cell lung cancer cells

**DOI:** 10.1186/s12931-021-01719-7

**Published:** 2021-05-12

**Authors:** Shaojia Wang, Chao Liu, Qing Lei, Zhengwei Wu, Xiangshuai Miao, Debing Zhu, Xu Yang, Na Li, Mingwei Tang, Yan Chen, Weiwei Wang

**Affiliations:** 1 Department of Gynecology, The Third Affiliated Hospital of Kunming Medical University, Yunnan Cancer Hospital, Yunnan Cancer Center, Kunming, 650118 China; 2 Department of Thoracic Surgery, The Third Affiliated Hospital of Kunming Medical University, Yunnan Cancer Hospital, Yunnan Cancer Center, No. 519 Kunzhou Road, Kunming, 650118 Yunnan China; 3 Department of Nuclear Medicine, The Third Affiliated Hospital of Kunming Medical University, Yunnan Cancer Hospital, Yunnan Cancer Center, Kunming, 650118 China; 4 Cancer Research Institute, The Third Affiliated Hospital of Kunming Medical University, Yunnan Cancer Hospital, Yunnan Cancer Center, Kunming, 650118 China

**Keywords:** NSCLC, Apoptosis, Gefitinib resistance, PCAT-1

## Abstract

**Background:**

Gefitinib, an epidermal growth factor receptor tyrosine kinase inhibitor, has been used as first-line treatment for advanced non-small-cell lung cancer (NSCLC). However, during treatment, cancer cells often develop resistance to gefitinib, the mechanisms of which are not fully understood. This study was designed to elucidate the expression and role of long non-coding RNA (lncRNA)-PCAT-1, a potential biomarker for drug resistance and a therapeutic target for NSCLC, in gefitinib resistance in NSCLC cells.

**Methods:**

In this study, we verified differential PCAT-1 expression in NSCLC gefitinib-resistant tissues or cells. PCAT-1 knockdown, clone formation, Transwell, flow cytometry, and immunofluorescence assays were used to verify the correlation between PCAT-1 and gefitinib sensitivity. A nude mouse tumor-bearing model verified that PCAT-1 can reverse gefitinib resistance in *vivo*. Then, a PI3K/Akt agonist was used to verify the possible mechanism of PCAT-1 action.

**Results:**

PCAT-1 is highly expressed in gefitinib-resistant NSCLC tissues and cells. PCAT-1 knockdown enhanced gefitinib sensitivity and gefitinib-induced apoptosis in H1299/GR cells. PCAT-1 knockdown reduced tumor volume and weight, and reversed acquired gefitinib resistance in vivo. PCAT-1 knockdown inhibited AKT and GSK3 phosphorylation in H1299/GR cells. A PI3K/AKT agonist reversed PCAT-1 knockdown-mediated enhancement of gefitinib sensitivity in H1299/GR cells

**Conclusion:**

PCAT-1 knockdown improves sensitivity to gefitinib by inhibition of AKT and GSK3 phosphorylation in NSCLC. PCAT-1 is as potential target for improving the clinical efficacy of gefitinib.

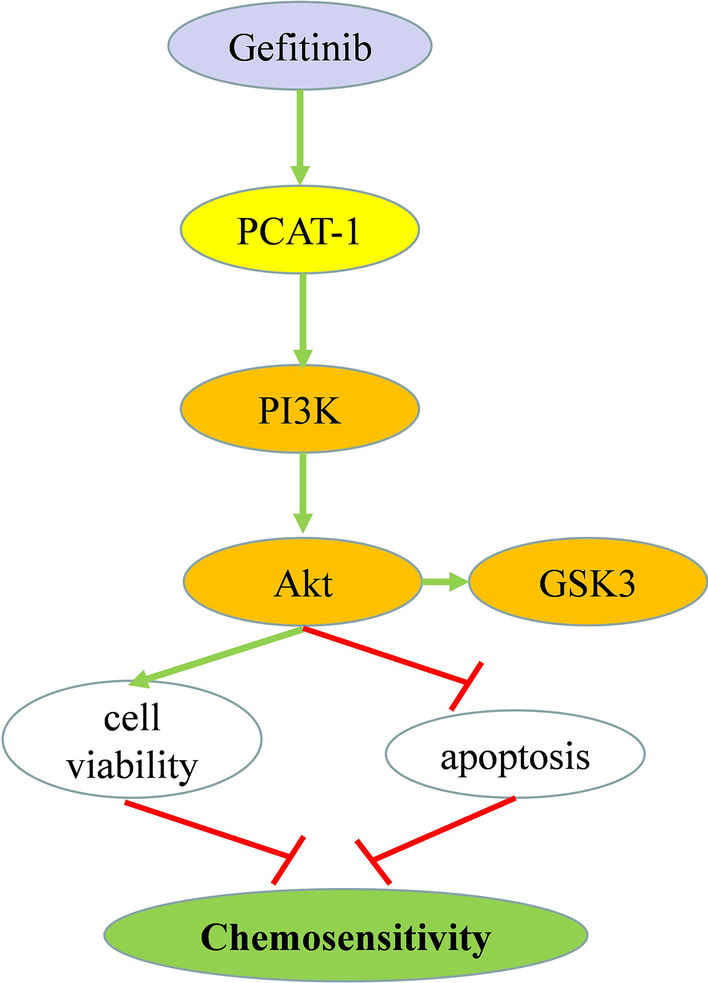

## Introduction

Lung cancer is the most common malignant tumor worldwide, and approximately 1.6 million people die from lung cancer every year [1]. In China, lung cancer incidence and mortality rank first among various malignant tumors [2]. When patients have chest tightness, shortness of breath, cough, weight loss, and other respiratory symptoms, most are already in the advanced stage of lung cancer. In such patients, it is difficult to achieve a radical cure with surgery and most treatments involve the use of radiotherapy or chemotherapy. At present, the median survival time of patients after first-line platinum-based chemotherapy is only 10 months [3]. The most common primary malignant tumor of the lung is epithelial cancer, which can be roughly divided into small cell carcinoma (SCLC) and non-small cell lung cancer (NSCLC) [4, 5]. Continuous breakthroughs have been made in the clinical diagnosis and treatment of lung cancer, greatly improving the survival rates of patients [6]. For example, gefitinib, the epidermal growth factor receptor (EGFR) tyrosine kinase inhibitor (TKI) for advanced lung cancer, is the first EGFR-TKI drug. Gefitinib has proven to be effective in preclinical studies. A variety of human cancer cell lines have anti-tumor activity [7–9]. Since being approved for first-line treatment, about 80% of patients with NSCLC with EGFR-positive mutations have significantly prolonged progression-free survival (PFS) after treatment with gefitinib [10]. However, in most patients, acquired gefitinib resistance occurs 6–12 months after treatment [11]. At present, the main mutations responsible for acquired gefitinib resistance are the T790M mutation, c-MET expansion, and PI3K/Akt signaling pathway activation [12]. Of these, the T790M mutation is the main cause, and greatly reduces the efficacy of EGFR-TKI therapeutic drugs [13]. Therefore, clarification of the TKI resistance mechanism in NSCLC is required to improve lung adenocarcinoma treatment.

Long non-coding RNAs (lncRNAs) are RNA molecules no longer than 200 nucleotides in length that are not translated into protein. lncRNAs regulate various biological processes, including apoptosis, proliferation, migration, and invasion [14, 15], and play important roles in chemotherapy and targeted therapy drug resistance [15]. lncRNAs play a crucial role in the progression, metastasis, prognosis, and drug resistance of NSCLC [16]. Recent studies have shown that lncRNA can also significantly increase drug resistance in targeted therapy [17, 18]. LINC00665 can interact with human epidermal growth factor receptor 2 (EZH2) and regulate the phosphatidylinositol 3-kinase (PI3K)/AKT pathway, indicating that it may be a potential biomarker of drug resistance and a therapeutic target for NSCLC [19]. The identification of additional lncRNAs involved in acquired gefitinib resistance will advance the development of drugs for targeted therapy and clinical application.

In this study, we examined the effect of the PCAT-1 lncRNA on gefitinib resistance. PCAT-1 is known to be related to drug resistance but has not been studied in NSCLC. The gefitinib-resistant H1299/GR cell line was used to explore the effect of PCAT-1 on gefitinib resistance and to elucidate its possible mechanism.

## Methods

### Clinical NSCLC specimens

Frozen non-small cell lung cancer tissues in control (no gefitinib treatment, n = 10) and gefitinib-resistant (relapsed after gefitinib treatment, n = 10) groups were collected from The Third Affiliated Hospital of Kunming Medical University. Our study was approved by the ethics committee of The Third Affiliated Hospital of Kunming Medical University, and informed consent was obtained from each patient before surgery.

### Cell culture

Lung adenocarcinoma H1299 cells were obtained from the Institute of Biochemistry and Cell Biology of the Chinese Academy of Sciences (Shanghai, China) and cultured RPMI-1640 media with 10% fetal bovine serum (FBS; Invitrogen, Carlsbad, CA, USA) at 37 °C with 5% CO_2_. Human lung epithelial BEAS-2B cells were incubated in DMEM medium containing 10% FBS, 100 U/ml penicillin, and 100 μg/ml streptomycin. All cells were passaged for 3 months or less before renewal from frozen early-passage stocks obtained from the indicated sources. Cells were tested to confirm that they were not contaminated with mycoplasma. Gefitinib (≥ 98%) was purchased from Santa Cruz Biotechnology (Santa Cruz, CA, USA). Gefitinib (8 μmol/L) in DMSO was diluted to the required concentrations in the growth media immediately before use. Gefitinib‐resistant H1299 cells were established from these cells by initially adding 1 μM gefitinib to the culture medium and, thereafter, increasing the dose to 100 μM.

### Cell transfection

According to the PCAT-1 sequence available in the National Center for Biotechnology Information database, the small interfering RNA specifically targeting PCAT-1 (si-PCAT-1) was constructed and transiently transfected into H1299 cells using Lipofectamine™ 2000 (Invitrogen, Carlsbad, CA, USA) according to the manufacturer's instructions. The pcDNA3.1 vector containing the blank sequence was used as a negative control (si-NC). Total RNA was isolated 48 h after transfection using TRIzol reagent (Dalian, China), and PCAT-1 expression levels were determined using qRT-PCR.

### Animals

All animal experiments were performed in accordance with The Third Affiliated Hospital of Kunming Medical University. Male nude mice (weight 18 ~ 22 g) were purchased from Shanghai SLAC Laboratory Animal Co., Ltd. (Shanghai, China) and housed in a dedicated room with 12 h dark/light cycle and controlled temperature (22 ± 1 °C). H1299/GR cells were transfected with si-NC or si-PCAT-1 and grown to 80% ~ 90% confluence. Trypsin (0.25%) was then added to separate and disaggregate cells. Cells were resuspended in phosphate-buffered saline (PBS) and the cell density adjusted to 1 × 10^7^ cells per ml. Each nude mouse was subcutaneously inoculated with 0.2 ml cell suspension in the right armpit. When the tumor volume grew to about 100 mm^3^, the nude mice that were successfully modeled were randomly divided into four groups: si-NC group, gefitinib group (gefitinib treatment after cell inoculation, 50 mg/kg·d), si-PCAT-1 group, and the gefitinib + si-PCAT-1 group. There were five mice in each group. Tumor volumes were measured every week for 2 months. Finally, the tumor tissue was removed, and the volume and weight were measured. Tumor volume was calculated based on caliper measurements using the following formula: tumor volume = ½ (length × width^2^) [20].

### RNA isolation and qRT-PCR analyses

SYBR Premix dimming eraser kit (TaKaRa, Dalian, China) was used for real-time quantitative RT-PCR detection on the ABI 7500 system (Applied Biosystems, Waltham, MA, USA). The primers were designed as follows: PCAT‐1 primers, forward: 5′‐AATGGCATGAACCTGGGAGGCG‐3′, reverse: 5-GGCTTTGGGAAGTGCTTTGGAG‐3′; and β-actin primers, forward: 5′‐CCATGTACGTTGCTATCCAGG‐3′, reverse: 5′‐TCTCCTTAATGTCACGCACGA‐3′. Primers were synthesized by Sangon (Shanghai, China). β-actin was used as an internal reference gene, and the mRNA value of the target gene was normalized by comparison with β-actin. The 2^−ΔΔCt^ method was used to calculate the relative fold change in target gene expression for performing statistical analyses [21].

### Cell viability

Cell Counting Kit-8 (Beyotime, Shanghai, China) was used for the CCK8 assay to determine the viability of each group of cells. In short, the cells were seeded in a 96-well plate at 5.0 × 10^3^/well and treated with gefitinib and si-NC or si-PCAT-1 at the specified concentration for 24 h. CCK8 reagent 10 µL was added to each well and incubated at 37 °C for 2 h. Then, absorption at 450 nm was evaluated using a microplate reader (Tecan, Männedorf, Switzerland).

### Colony formation assay

For the colony formation assay, H1299/GR cells were spread evenly in a 6-well plate (300 cells per well). Twelve hours later, si-NC and si-PCAT-1 plasmids were transfected into the cells. At the end of the experiment, cells were fixed with ice-cold methanol (Dalian Meilun Biotechnology Co., Ltd., Dalian, China), stained with 0.1% crystal violet (Sigma, St. Louis, MO, USA), and colonies were counted. Colonies containing at least 20 regions were scored, and data were given as colony number per 6-well plate.

### Transwell assay

H1299/GR cells were transfected with si-NC and si-PCAT-1 for 48 h. After 48 h of cell transfection, 50,000 cells diluted in serum-free medium were seeded into the upper chamber (8.0 μm, Millipore, Burlington, NJ, USA) with or without Matrigel coating (Sigma) for Transwell invasion or migration assays, respectively [22]. The upper chamber was placed in the lower chamber of a medium containing 10% fetal bovine serum for 24 h. The cells on the upper were gently scraped and rinsed using a cotton swab. The lower surface was immersed in the cell fixation solution for 30 min and stained with 0.05% crystal violet for 2 h. Microscopy was used to photograph pictures of five random areas in each insert, and the number of cells that had reached the underside of the inserts was counted. This experiment was independently repeated three times.

### Flow cytometry analysis

#### Apoptosis analysis

The H1299/GR cells (4 × 10^4^ cells per well) were seeded in a 12-well plate for flow cytometry analysis of apoptosis. After 12 h of si-PCAT-1 or si-NC transfection, each well was replaced with cell culture medium with gefitinib or DMSO and incubated for another 72 h. The AnnexinV-FITC/PI Apoptosis Kit (MultiSciences Biotech, China) was used to measuring apoptosis. Flow cytometry analysis was performed in the BD FACSCalibur™ (BD Bioscience, USA) flow cytometer system.

#### Cell cycle analysis

H1299/GR cells were seeded into 6-well culture plates and allowed to grow overnight. After transfection with si-PCAT-1 or si-NC, the cells were treated with 8 μmol/L of gefitinib. DMSO was included as a control. After the treatments, cells were collected and fixed with 70% ethanol, stained with propidium iodide, and analyzed by flow cytometry with 5 × 10^4^ events counted per run. The proportion of cells in the G1, S, and G2/M phases of the cell cycle was detected using FlowJo software (FlowJo, Ashland, OR, USA).

### Immunofluorescence

Immunofluorescence experiments were used to stain and observe caspase 3 in each cell group. First, the cells crawled over the culture plate were washed with PBS three times, for 3 min each. Paraformaldehyde (4%) was used to fix the cells for 15 min. Then, the slides were washed three times with PBS for 3 min each time. Cells were incubated with 0.5% Triton X-100 (prepared in PBS) at room temperature for 20 min, and then normal goat serum was dropped on the slide to block non-specific antigens. Excess liquid was absorbed using absorbent paper, diluted caspase 3 primary antibody (1:200, ab13847, Abcam, USA) was added to each slide. The slides were placed in a humidified box and incubated overnight at 4 °C. The next day, the corresponding fluorescent secondary antibody was dropped on the glass slide, and DAPI was added in the darkroom for 5 min to stain the nucleus. Excess DAPI was washed away by four 5 min washes with PBST. A fixative containing an anti-fluorescence quencher was added to the glass slide, and the image was observed and collected under a fluorescence microscope.

### Immunohistochemistry

Lung cancer tissues isolated from nude mice were fixed with 4% paraformaldehyde and cut into 5 μm paraffin sections. After routine rehydration and antigen retrieval, goat serum was dripped onto the slide to block non-specific antigens. Diluted primary antibodies, Akt (1:1000, Ab8805, Abcam) and p-Akt (1:50, Ab38449, Abcam) were added to the slides, and the slides incubated overnight in a humidified chamber at 4 °C. The biotinylated secondary antibody working solution was added to the glass slides. After the antibody incubation, the slides were washed four times in PBS for 5 min each time. HRP-labeled streptavidin working solution was added to the slices, and the slices were placed in a humid chamber at room temperature for 20 min. Freshly prepared DAB coloring solution was dropped onto the slices and sealed with neutral glue. The staining on the slide was observed under the microscope and pictures were taken.

### Western blot analysis

RIPA lysis buffer (Beyotime, China) was used to extract protein from cells or tissues, and protein concentration was measured using the Bradford analysis (BioRad, USA). The same amount of protein (20 µg/lane) was separated by sodium dodecyl sulfate–polyacrylamide gel electrophoresis (SDS-PAGE, 8%–12%) and transferred to a PVDF membrane (Millipore, USA). Then, the membrane was blocked using 5% (w/v) skimmed milk at room temperature for 1 h. The membrane was incubated with Akt (1:500, Ab8805), p-Akt (1:500, Ab38449), PI3K (1:2000, Ab140307), GSK3 (1:5000, Ab40870), p-GSK3 (1:10,000, Ab75814), and β-actin (1:1000, Ab6267) primary antibodies overnight at 4 °C. After which, the membrane was incubated with secondary antibody at 37 °C for 2 h. The enhanced chemiluminescence reaction mixture was dropped onto the film and reacted in a dark room for 2–3 min. A specific band was detected using the ECL system (Amersham, Buckinghamshire, UK). ImageJ software was used quantify band intensity.

### Statistic analysis

Experiments requiring statistical analysis were repeated three times. Experimental data were presented as mean ± standard errors. Statistical analysis was performed using SPSS software (version 13.0, SPSS, USA) and a one-way ANOVA. A significant difference was declared if P < 0.05.

## Results

### Relative PCAT-1 expression in NSCLC tissues and cell lines

**Fig. 1:**
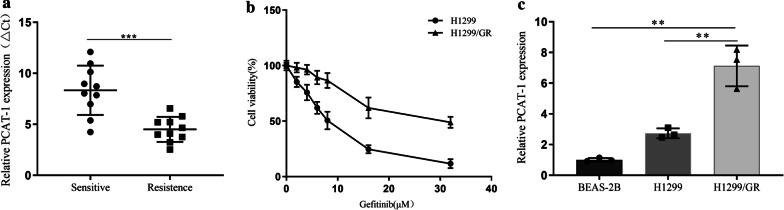
Relative PCAT-1 expression in non-small cell lung cancer (NSCLC) tissues and cell lines. **a** PCAT-1 expression in NSCLC tissues from patients who had never been treated with gefitinib and in patients treated with gefitinib but who developed resistance was measured by qRT-PCR. **b** H1299 and H1299/GR cell viability was measured by CCK8 assay after treatment with various concentration of gefitinib for 24 h. **c** PCAT-1 expression was analyzed by qRT-PCR in human lung epithelial BEAS-2B cells, gefitinib-sensitive H1299 cells, and gefitinib resistant H1299 cells. *, p < 0.05, **, p < 0.01

PCAT-1 expression was detected in NSCLC tissue samples that have never used gefitinib, and in samples resistant to gefitinib. The results showed that PCAT-1 expression was significantly higher in the drug-resistant group (Fig. 1a). To further uncover the role of PCAT-1 in NSCLC resistance, we used H1299 cells to construct the H1299/GR gefitinib-resistant cell line (Fig. 1b). PCAT-1 expression was detected in H1299/GR cells. The results show that PCAT-1 expression was significantly higher in H1299/GR cells than in MCR-5 and H1299 cells (Fig. 1c).

### PCAT-1 knockdown mediated gefitinib sensitivity in GR cells

**Fig. 2:**
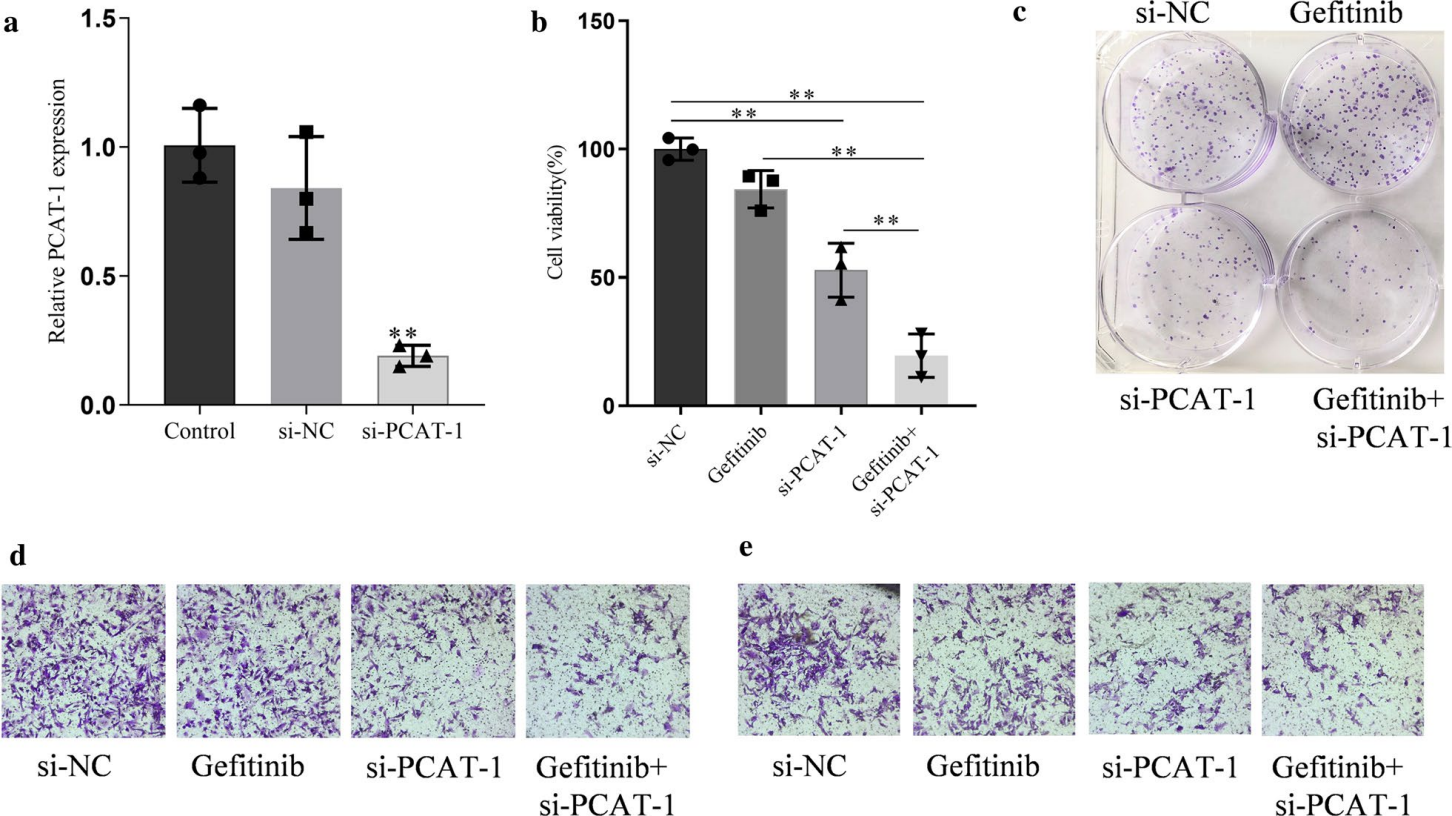
PCAT-1 knockdown led to gefitinib sensitivity in gefitinib resistant (GR) cells. **a** The efficiency of siRNA-mediated PCAT-1 knockdown was measured by qRT-PCR. **b** Cell viability was measured by CCK8 assay in H1299/GR cells transfected with si-NC or si- PCAT-1 after gefitinib treatment for 24 h. **c** Colony formation assays were performed to measure proliferation in GR cells transfected with si-NC or si- PCAT-1 after gefitinib treatment. **d** Transwell assays were performed to observe the migration of GR cells transfected with si-NC or si- PCAT-1 after gefitinib treatment. **e** Transwell assays were performed to observe the invasion of GR cells transfected with si-NC or si- PCAT-1 after gefitinib treatment. *, p < 0.05, **, p < 0.01

PCAT-1 expression was knocked-down by RNA interference to further study its role in gefitinib resistance. PCAT-1 expression was significantly reduced in cells transfected with si-PCAT-1, but there si-NC transfection did not affect PCAT-1 expression (Fig. 2a). Cell viability results showed that after knocking down PCAT-1, the viability of gefitinib + si-PCAT-1 group cells was significantly reduced, but the viability of gefitinib alone group cells did not differ from that of the si-NC group (Fig. 2b). The results of clone formation experiments showed that after PCAT-1 knockdown, the cell proliferation ability was significantly reduced (Fig. 2c). The invasion and migration abilities of H1299/GR cells were tested using Transwell assays. The results showed that the invasion and migration abilities of H1299/GR cells were significantly reduced after PCAT-1 knockdown, but there was no difference between gefitinib and si-NC groups (Fig. 2d, e).

### PCAT-1 knockdown enhanced gefitinib-induced apoptosis in H1299/GR cells

**Fig. 3:**
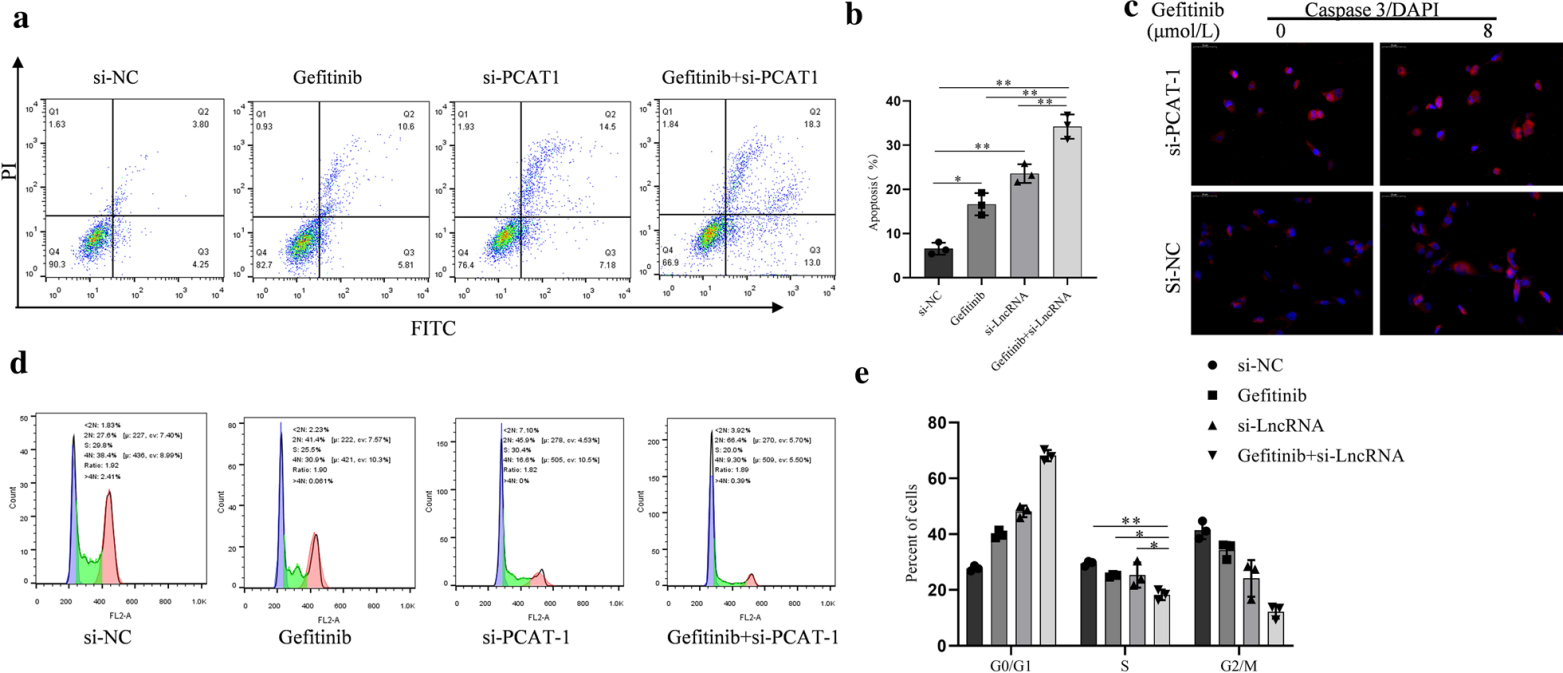
PCAT-1 knockdown enhanced gefitinib-induced apoptosis in H1299/GR cells. **a**, **b** Flow cytometric analysis was performed to detect the percentage of apoptotic cells. **c** PCAT-1 Knockdown enhanced cleaved Caspase 3 expression, as measured by immunofluorescence. The cell cycle distribution of H1299/GR cells were analyzed using flow cytometry after transfection. *, p < 0.05, **, p < 0.01

Various experiments have been used to verify whether PCAT-1 has a regulatory effect on gefitinib-induced apoptosis. Apoptosis detection by flow cytometry showed that PCAT-1 knockdown significantly increased the number of apoptotic cells after treatment with gefitinib, while there was no difference between si-NC and gefitinib groups (Fig. 3a, b). Immunofluorescence detection of caspase-3 showed that PCAT-1 regulates gefitinib-induced apoptosis promotion. PCAT-1 knockdown significantly reduced the caspase-3 fluorescence intensity (Fig. 3c). Flow cytometry analysis of cell cycle distribution showed that after PCAT-1 knockdown, the proportion of cells in S phase was significantly reduced, but no difference was observed between si-NC and gefitinib groups. This result shows that cell cycle progression was significantly hindered in H1299/GR after PCAT-1 knockdown (Fig. 3d, e).

### PCAT-1 knockdown reverses acquired resistance to gefitinib in vivo

**Fig. 4:**
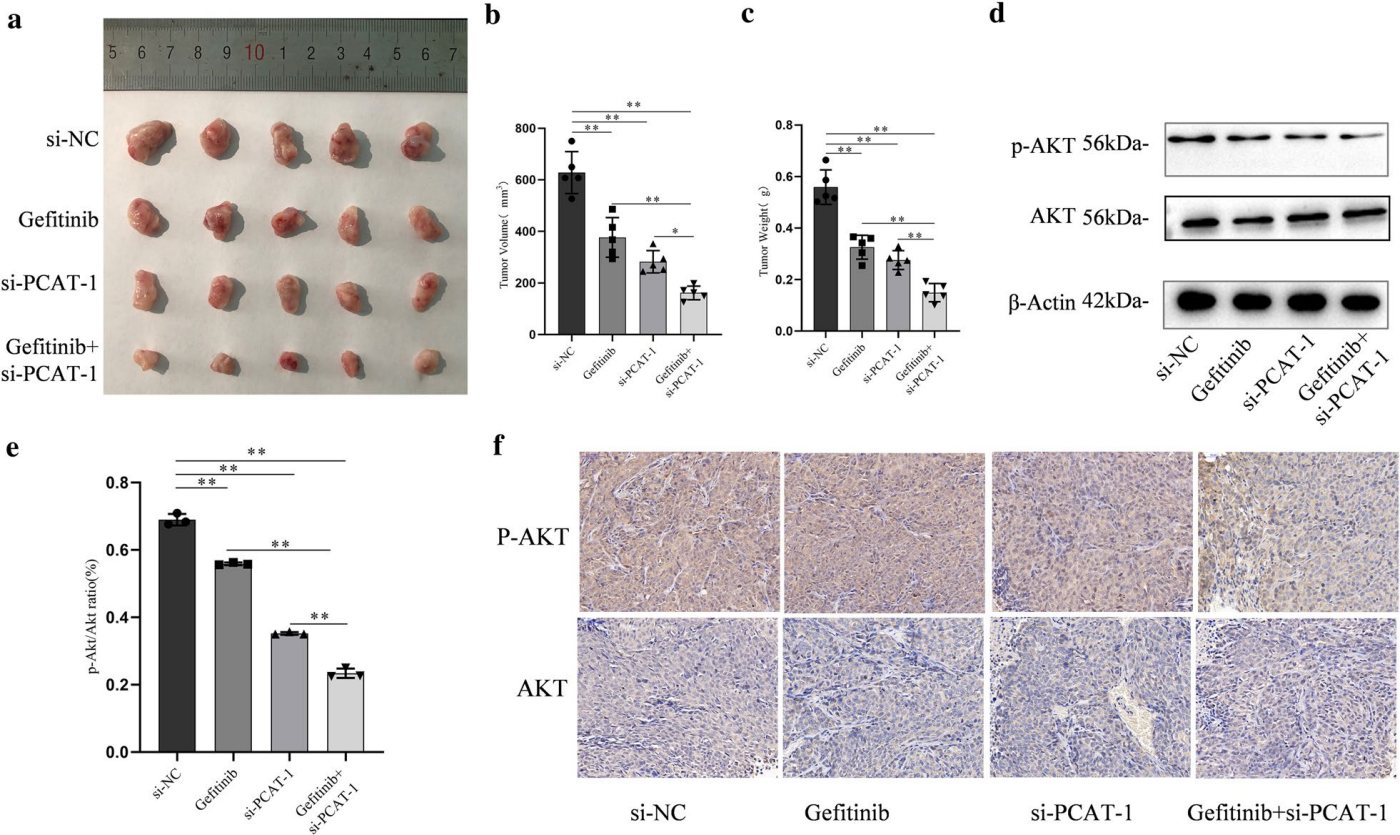
PCAT-1 knockdown reverses acquired resistance to gefitinib in vivo. **a** PCAT-1 knockdown enhanced gefitinib-induced growth inhibition in xenograft tumors **b** Tumor volume was measured every 5 days. **c** Tumor weight was measured after removal. qRT-PCR was used to detect p-AKT (**d**) and AKT (**e**) expression. **f** The tumor sections were examined using immunohistochemical staining with AKT or p-AKT antibodies. *, p < 0.05, **, p < 0.01

Subcutaneous tumor-bearing nude mice were used to verify the regulatory effect of PCAT-1 on gefitinib resistance in vivo. After transfecting experiments and transplanting H1299 cells them under the skin of mice, lung cancer tumor tissue was successfully formed (Fig. 4a). The volume and weight of the subcutaneous tumors were measured and it was found that after gefitinib was administered alone, tumor volume and weight were significantly reduced compared to those in the controls. Tumor volume and weight were further reduced after knockout PCAT-1 (Fig. 4b, c). To further study the mechanism of PCAT-1, we examined the AKT/p-Akt apoptosis-related protein. PCAT-1 knockdown significantly reduced the activity of Akt activity and inhibited Akt-mediated apoptosis (Fig. 4d, e). The results of immunohistochemistry experiments also showed that PCAT-1 knockdown can reduce Akt phosphorylation (Fig. 4f).

### PCAT-1 knockdown mediates gefitinib sensitivity through targeting the PI3K/AKT pathway

**Fig. 5:**
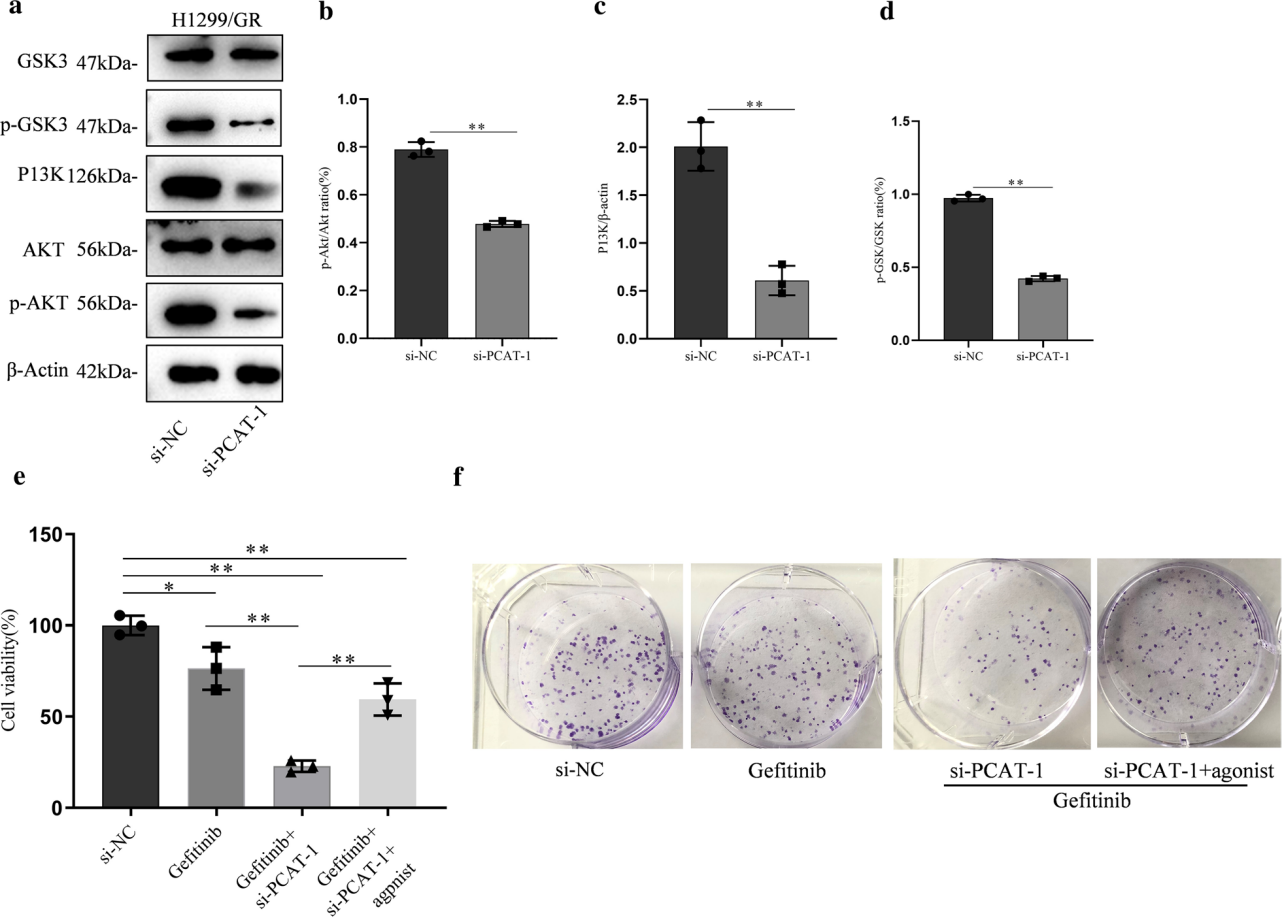
PCAT-1 knockdown mediates gefitinib sensitivity through targeting the PI3K/AKT pathway. **a** The effects of PCAT-1 knockdown on the PI3K/Akt pathway. PCAT-1 knockdown inhibited AKT (**b**), P13K (**c**), and GSK3 (**d**) phosphorylation in H1299/GR cells. PCAT-1 knockdown-mediated gefitinib sensitivity was reversed in H1299/GR cells after PI3K/AKT agonist treatment, as measured by CCK8 (**e** and colony formation assays (**f**). *, p < 0.05, **, p < 0.01

The mechanism by which PCAT-1 regulates of gefitinib resistance was further verified in H1299/GR cells. After PCAT-1 knockdown, Akt and GSK phosphorylation and PI3K expression were significantly reduced (Fig. 5a–d). AKT agonists were used to assess whether PCAT-1 regulates gefitinib resistance through the AKT pathway. Cell viability experiments showed that cell viability was significantly reduced after PCAT-1 knockdown, compared to control groups. PCAT-1 knockdown and AKT agonist administration significantly increased cell viability (Fig. 5e). The results of clone formation experiments revealed the same trend. In PCAT-1 knockdown cells, the proliferation ability was significantly lower than that of the control groups. Cell proliferation ability was significantly increased in cells with PCAT-1 knockdown that were treated with AKT agonist (Fig. 5f).

## Discussions

This study first found that PCAT-1 was highly expressed in clinical NSCLC resistant tissues and gefitinib resistant cells. Through cell viability, cell proliferation, and apoptosis experiments, we found that PCAT-1 knockdown can significantly increase the sensitivity of gefitinib to H1299/GR cells and increase the apoptosis caused by gefitinib. Tumor-bearing experiments in nude mice confirmed that this regulatory effect also occurs in vivo. The results of AKT agonist experiments confirmed that PCAT-1 regulates gefitinib resistance through the PI3K/Akt pathway. Our results provide a potential solution for improving the clinical resistance of gefitinib.

Gefitinib is the first EGFR-TKI drug. In preclinical studies, gefitinib has anti-tumor activity against various human cancer cell lines expressing EGFR, including lung, ovarian, breast, and colon cancer [23]. However, the efficacy of gefitinib is challenged by drug resistance. The main resistance mechanism includes acquired resistance that relies on the EGFR pathway. The traditional view is that the threonine at the 790th amino acid (T790M) of EGFR exon 20 is replaced by methionine, which increases the affinity of ATP for EGFR, which weakens the binding effect of gefitinib [24, 25]. Shikonin significantly inhibits EGFR phosphorylation and leads to EGFR degradation, which promotes recovery of gefitinib sensitivity in gefitinib-resistant NSCLC cells with T790M and L858R activation mutations [26]. Other EGFR mutations include L792X, G796S, L718Q, G 724S, and exon 20 insertion mutations [27]. Wu et al. discovered a new type of dual inhibitor of EGFR and hepatocyte growth factor receptor (cMET), N19, which simultaneously degrades two proteins through the ubiquitin–proteasome, effectively overcoming gefitinib resistance in EGFR mutant NSCLC cells [28]. The mechanism of acquired gefitinib resistance caused by the independent EGFR pathway involves transmembrane tyrosine kinase receptor (MET) amplification/hepatocyte growth factor (HGF) overexpression [29], and AXL/GAS6 activation that leads to MEK/ERK and PI3K/AKT pathway activation [30].

To combat gefitinib resistance, combining strategies including gene therapy, drug combination, and drug repurposing has become a focus of research [31, 32]. A study collected newly-treated patinates with NSCLC and EGFR gene mutations in East Asia and randomly divided them into gefitinib combined with pemetrexed (n = 126) and simple oral gefitinib (n = 65) groups. Their results showed that combination therapy can significantly increase PFS (P = 0.029) and delay disease progression [33].

Much progress has also been made in molecular biology. Studies have found that senescence promoted by autophagy contributes to gefitinib resistance, and autophagy inhibition enhances apoptosis and eliminates senescence [34]. PTEN promoter CpG hypermethylation leads to decreased PTEN expression [35]. Inhibition of miR-873 increases the resistance of NSCLC cells to gefitinib by upregulating GLI1 [36]. However, the problem of drug resistance still exists [37, 38]. Therefore, the use of epigenetics to discover mechanisms to improve the sensitivity of targeted drugs is of great significance to the emergence of new drug resistance in the future.

lncRNA, as a non-coding RNA, has roles in the proliferation, migration, and apoptosis of tumor cells, which has important research value. The role of lncRNAs in NSCLC has been confirmed. For example, HOXA-AS3 confers cisplatin resistance by down-regulating the expression of homeobox A3 (HOXA3), and HOXA3 knockdown can increase cisplatin resistance [39]. lncRNA UCA1 and lncRNA BC087858 can also promote gefitinib resistance [18, 40]. PCAT-1, a lncRNA related to stress resistance, plays a role in drug resistance in a variety of cancers. PCAT-1 confers DDP resistance in gastric cancer (GC) cells through the miR-128/ZEB1 axis, providing a promising therapeutic strategy for GC [41]. PCAT1 levels are elevated in ccRCC tumors and several ccRCC cells, and PCAT1 knockdown with siRNA (si-PCAT1) alleviated Caki-2 and ACHN cell proliferation, migration, and invasion [42]. PI3K/AKT and extracellular signal-regulated protein kinases 1 and 2 (ERK 1/2) are two key signal pathways involved in cell signal transduction and tumor cell survival and proliferation [43]. This is used by the SDF-1/CXCR4 axis to inhibit apoptosis and promote tumor cell growth and proliferation [44]. Our results show that PCAT-1 enhances the proliferation and pro-apoptosis effects of gefitinib in NSCLC cells by reducing AKT phosphorylation.

Our clinical sample, cell experiment, and animal experiment results show that PCAT-1 improves NSCLC cell sensitivity to gefitinib. However, limited resources and funds mean that a small number of clinical samples were used in this study, which casts a shadow over the reliability of clinical results. Additionally, the specific roles of molecules acting downstream of PCAT-1 have not been clarified, and only the effect of PCAT-1 on the PI3K/Akt pathway has been confirmed. Therefore, future studies should investigate more detailed mechanisms through which PCAT-1 improves gefitinib resistance and include a larger number of clinical samples.

We have shown, for the first time, that PCAT-1 knockdown can inhibit proliferation and apoptosis in NSCLC H1299/GR cells treated with gefitinib and inhibit tumor formation in vivo. PCAT-1 knockdown improves sensitivity to gefitinib by inhibiting AKT and GSK3 phosphorylation in NSCLCs. Therefore, PCAT-1 may be an effective target to improve the effect of gefitinib effect in the treatment of NSCLC.

## Data Availability

All data analyzed during the study are presented in the main manuscript. The data that support the findings of this study are available from the corresponding author upon reasonable request.

## Abbreviations

NSCLC: Non-small-cell lung cancer
lncRNA: Long non-coding RNA
PCAT-1: Prostate cancer associated transcript-1
PI3K: Phosphatidylinositol-3-kinase
SCLC: Small cell carcinoma
EGFR: Epidermal growth factor receptor
TKI: Tyrosine kinase inhibitor
PFS: Progression-free survival
EZH2: Epidermal growth factor receptor 2
PBS: Phosphate-buffered saline
Cmet: Hepatocyte growth factor receptor
HGF: Hepatocyte growth factor
HOXA3: Homeobox A3
GC: Gastric cancer
ERK 1/2: Extracellular signal-regulated protein kinases 1 and 2
